# Identification of ischemic stroke subtypes defined by inflammation, coagulation, and metabolic profiles

**DOI:** 10.3389/frai.2026.1776891

**Published:** 2026-03-10

**Authors:** Hezhen Gao, Dilraba Mahmut, Fanshu Dai, Haimiao Yu, Wei Chang, Xingya Huang, Biao Zhang

**Affiliations:** Tianjin Key Laboratory of Cerebral Vascular and Neurodegenerative Diseases, Department of Clinical Laboratory, Huanhu Hospital Affiliated to Tianjin Medical University, Tianjin, China

**Keywords:** clustering analysis, coagulation, inflammation, ischemic stroke, metabolic profiles

## Abstract

**Background:**

Ischemic stroke is a heterogeneous disease influenced by inflammation, coagulation dysfunction, and metabolic disturbances. However, integrated analysis incorporating these biological domains for patient stratification remain limited.

**Methods:**

A retrospective study of 132 ischemic stroke patients was conducted. Clinical, coagulation, inflammatory, and metabolic parameters were collected. Principal component analysis (PCA) was applied for dimensionality reduction and visualization. *K*-means clustering was then used to identify subtypes with the optimal cluster number validated by elbow plot and silhouette analysis. Differences among cluster’s groups were assessed using ANOVA or Kruskal–Wallis tests for continuous variables and Chi-square tests for categorical variables.

**Results:**

PCA revealed underlying heterogeneity among patients. Validated *K*-means clustering identified three distinct subtypes. Cluster 1 represented a low inflammatory subtype with reduced inflammatory markers. Cluster 2 was a high inflammatory and hypercoagulable subtype, characterized by elevated WBC, NEU, hsCRP, FIB, D-dimer, PT, INR along with a higher prevalence of coronary heart disease and carotid plaque, smoking, and drinking. Cluster 3 was a metabolic risk subtype, characterized by relatively younger age, elevated TG, CHOL, HDL-C, LDL-C, APOB, APOA-1 and APOB/APOA1 ratio, and intermediate inflammatory activity.

**Conclusion:**

Data driven clustering identified biologically distinct ischemic stroke subtypes based on inflammation, coagulation, and metabolic profiles. This stratification highlights the heterogeneity of ischemic stroke and may inform future personalized approaches to risk assessment and management.

## Introduction

Stroke remains one of the leading causes of long-term disability and mortality worldwide, representing a substantial public health burden despite advances in prevention and acute management strategies. According to the Global Burden of Disease Study, Ischemic stroke (IS) represents the predominant stroke subtype and is a leading cause of premature mortality and disability worldwide (Collaborators GBDS, 2021). While traditional risk factors such as hypertension, diabetes mellitus, and dyslipidemia are well established, considerable heterogeneity exists in clinical presentation, pathophysiological mechanisms, and prognosis among IS patients (Campbell and Khatri, 2020). This heterogeneity underscores the need for refined approaches to classify patients into biologically and clinically meaningful subgroups.

Growing evidence highlights the pivotal role of inflammation, coagulation dysfunction, and metabolic disturbances in the pathogenesis and progression of IS. Inflammation contributes to all stages of stroke, from the initiation of atherosclerosis to post-ischemic injury (Denorme et al., 2022; Lombardozzi et al., 2025). Elevated inflammatory biomarkers such as high-sensitivity C-reactive protein (hsCRP) and white blood cell (WBC) count have been consistently associated with stroke incidence and poorer outcomes (Esenwa and Elkind, 2016; Luna et al., 2014). Similarly, hypercoagulability, characterized by abnormalities in prothrombin time (PT), fibrinogen (FIB), and D-dimer, is strongly linked to thrombus formation and recurrent ischemic events (Spiegelenberg et al., 2025; Zhang et al., 2021). In parallel, dysregulated lipid and glucose metabolism, reflected by elevated triglycerides (TG), low density lipoprotein cholesterol (LDL-C), and impaired glucose tolerance, has been shown to influence stroke risk and recovery processes (Amarenco et al., 2020).

Despite accumulating evidence, the extant literature has largely concentrated on single biomarkers or isolated pathways, thereby insufficiently capturing the multifaceted heterogeneity of stroke. Studies that individually evaluate the prognostic utility of fibrinogen or high sensitivity C-reactive protein provide valuable yet intrinsically limited insights (Hou et al., 2021; Mccabe et al., 2021). Similarly, studies focused on lipid profiles or glucose metabolism have delineated associations with stroke susceptibility, but seldom incorporate these findings with coagulation and inflammatory indices within a unified analytical framework (Liu et al., 2025). Such reductionist strategies overlook the dynamic interplay among inflammation, coagulation, and metabolic dysfunction, which are biologically interdependent and jointly precipitate both the incidence and the clinical process of cerebrovascular events.

Recent advances in data driven methodologies, such as principal component analysis (PCA) and unsupervised clustering, offer novel opportunities to unravel complex interactions among multiple biomarkers. Clustering techniques enable the identification of patient subgroups that share distinct clinical and biochemical signatures, thereby revealing hidden patterns not detectable through traditional statistical methods (Angelini et al., 2022; Bernabe-Diaz et al., 2025). In cardiovascular medicine, unsupervised clustering has successfully delineated subtypes of heart failure and myocardial infarction with important prognostic and therapeutic implications (Shah et al., 2015). However, the application of such approaches in ischemic stroke remains limited, and few studies have attempted to define subgroups of stroke patients based on an integrated analysis of inflammatory, coagulation, and metabolic profiles.

The present study addresses this knowledge gap by applying unsupervised clustering to a cohort of IS patients. By simultaneously evaluating clinical characteristics and a wide spectrum of laboratory markers, including coagulation indices, inflammatory biomarkers, and lipid metabolism parameters. We aim to identify biological and clinically relevant IS subtypes. We hypothesize that this approach will uncover distinct patient groups with differential risk profiles, which may ultimately inform individualized prevention strategies and tailored treatment approaches. Our study thus provides a comprehensive, data driven framework for identifying biologically distinct IS subtypes, with potential implications for future precision medicine approaches.

## Materials and methods

### Study population

This retrospective study enrolled a total of 132 patients diagnosed with acute ischemic stroke who were admitted to the Department of Neurology at Huanhu Hospital affiliated to Tianjin Medical University between April 2024 and May 2025. The diagnosis of ischemic stroke was confirmed by clinical evaluation and neuroimaging (MRI or CT), according to the Chinese guidelines for diagnosis and treatment of acute ischemic stroke 2014 (Chinese Society of Neurology and Chinese Stroke Society, 2016). All patients were age ≥18 years, a confirmed diagnosis of ischemic stroke within 7 days of symptom onset, and availability of complete clinical and laboratory data. Exclusion criteria included hemorrhagic stroke, transient ischemic attack, or cerebral venous thrombosis; the presence of systemic infection, autoimmune disease, or hematological malignancy at admission; chronic liver or renal insufficiency; active infection and current use of anticoagulants or immunosuppression therapy. The study protocol was reviewed and approved by the Ethics Committee of Tianjin Huanhu Hospital (Approval No. 2020–6).

Despite the potential for larger retrospective cohorts, our sample size was constrained by stringent inclusion/exclusion criteria aimed at minimizing confounding factors. This allowed us to focus on a relatively homogeneous cohort with complete biomarker data.

### Data collection

Data for this retrospective cohort study were systematically collected from the electronic medical record (EMR) system and the laboratory information system (LIS) of Huanhu hospital affiliated to Tianjin Medical University. The demographic and clinical data were extracted from the structured and unstructured fields of the EMR, including age, sex, body mass index (BMI), drinking and smoking status, and medical history of hypertension, diabetes, coronary artery disease, history of previous stroke, carotid plaque as well as National Institutes of Health Stroke Scale (NIHSS) score at admission.

Laboratory parameters were obtained within 24 h of symptom onset. Hematological, coagulation, metabolic, and inflammatory biomarkers were assayed using standardized automated platforms, with data retrieved directly from the LIS to ensure integrity.

### Statistical analysis

All statistical analyses were performed using R software (version 4.5.1). To reduce dimensionality and visualize variance across patients, principal component analysis (PCA) was applied to the standardized dataset. *K*-means clustering was then used to classify patients into subgroups based on their combined clinical, inflammatory, coagulation, and metabolic profiles. To objectively determine the optimal number of clusters *k*, we performed elbow method and silhouette analysis. The elbow method plots the total within-cluster sum of squares against *k*, with the optimal *k* indicated by an inflection point where the rate of decrease sharply changes. Silhouette analysis evaluates both cluster cohesion and separation, with higher average silhouette widths indicating better-defined clusters. Both analyses converged on *k* = 3 as the optimal number (see Supplementary Figure S1), which was subsequently used for *K*-means clustering. Clustering was performed on the full standardized dataset to retain all biomarker information, PCA was used solely for visualization. Heatmaps were generated to visualize the overall patterns of variable distribution across the identified clusters, providing a broad view of the biological profiles of each cluster. Quantitative comparisons of individual biomarkers are presented with pairwise comparisons and scatter plots of individual.

Continuous variables were tested for normality using the Shapiro–Wilk test, and all data were expressed as median (range). Intergroup differences of normally distributed variables were compared using one-way analysis of variance (ANOVA). Comparisons of non-normally distributed variables were conducted using the Kruskal–Wallis test. Bonferroni and Mann–Whitney test were applied to pairwise comparisons between groups for normal and non-normal distributions, respectively. Categorical variables were expressed as counts and percentages, and differences among groups were assessed using the Chi-square test or Fisher’s exact test as appropriate. A two-tailed *p* < 0.05 was considered statistically significant.

## Results

### Principal component analysis (PCA) and *K*-means clustering

PCA was performed to reduce dimensionality and visualize the overall variance patterns in the dataset. The first two principal components (PC1 and PC2) explained 11.6 and 9.9% of the total variance, respectively. While the two-dimensional PCA visualization showed partial separation among patients, it primarily reflected underlying heterogeneity rather than definitive cluster boundaries.

To further confirm subgroup structure, unsupervised clustering *K*-means algorithm was applied to the full standardized dataset. Objective validation of the cluster number was performed prior to *K*-means clustering. Both elbow method and silhouette analyses supported the choice of *K* = 3 as the optimal number of clusters, with the elbow plot showing a clear inflection point and silhouette scores having a second peak at *K* = 3 (Supplementary Figure S1). This validated *K* was then used in the subsequent *K*-means algorithm.

**Figure 1:**
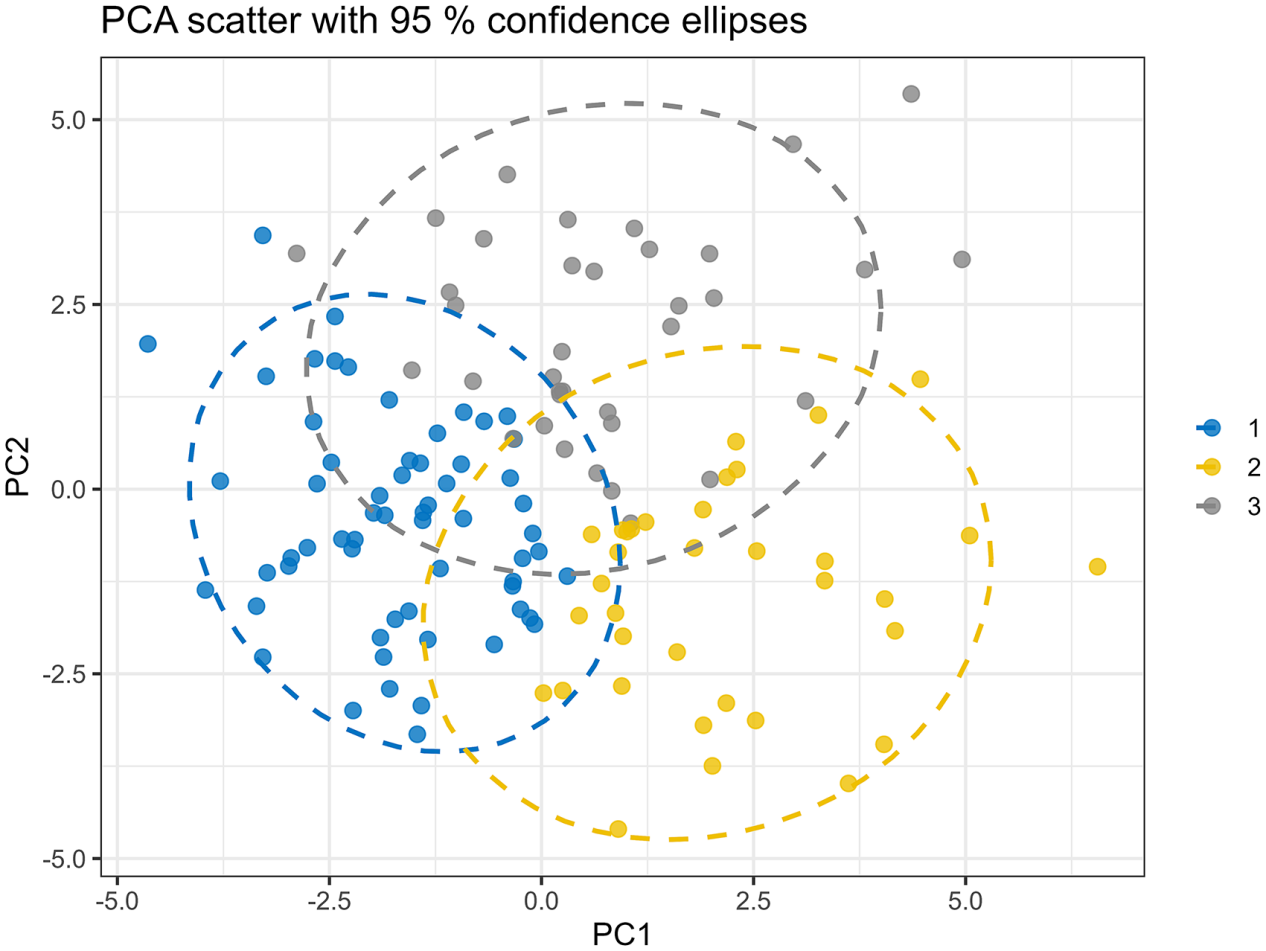
Identification of stroke patient subtypes via PCA and *K*-means clustering analysis. PCA was used for visualization of variance structure, while *K*-means clustering was conducted on the full standardized dataset. The projection onto the first two principal components is shown for illustrative purposes only. Scatter plot of the first two principal components (PCs) derived from 40 variables across 132 stroke patients, with all variables *Z*-score standardized prior to analysis. Each point represents an individual patient, positioned according to their scores on PC1 and PC2. Points are colored based on *K*-means clustering results.

Based on the *K*-means algorithm, *K*-means clustering identified three heterogeneity patient subgroups characterized by distinct inflammatory, coagulation, and metabolic profiles (Figure 1). Importantly, *K*-means clustering was performed on the full standardized dataset rather than being restricted to the first two principal components, thereby retaining the complete multivariate information. Together, PCA visualization and clustering analysis supported the presence of multidimensional heterogeneity within this cohort.

**Figure 2:**
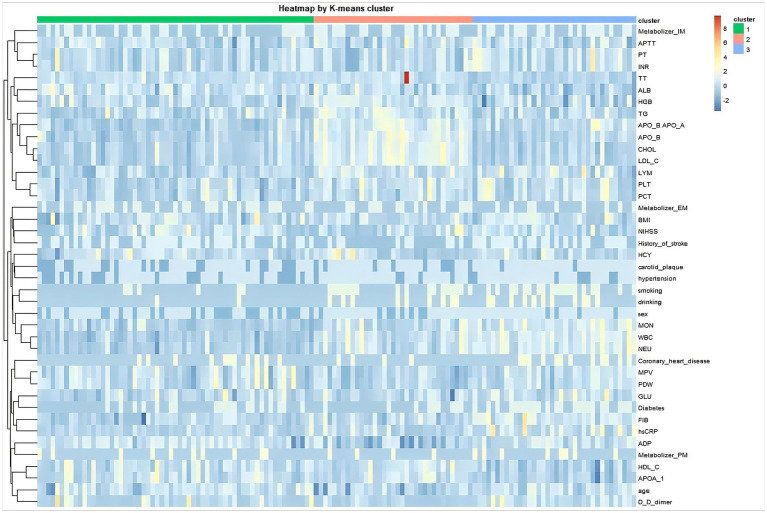
Heatmap of clinical and laboratory characteristics in stroke patients based on *K*-means clustering. This visualization differentiates three patient subtypes with distinct clinical and laboratory profiles. The heatmap is intended to illustrate overall clustering patterns across multidimensional laboratory variables rather than to convey precise quantitative differences between individual biomarkers. Columns represent individual patients, grouped into three subtypes by *K*-means clustering. Rows represent clinical and laboratory variables, which were hierarchically clustered to group variables with similar patterns. Dendrograms on the top and left illustrate the hierarchical clustering relationships among patients and variables, respectively. The annotation bar adjacent to the heatmap indicates the *K*-means cluster assignment for each patient.

**Figure 3:**
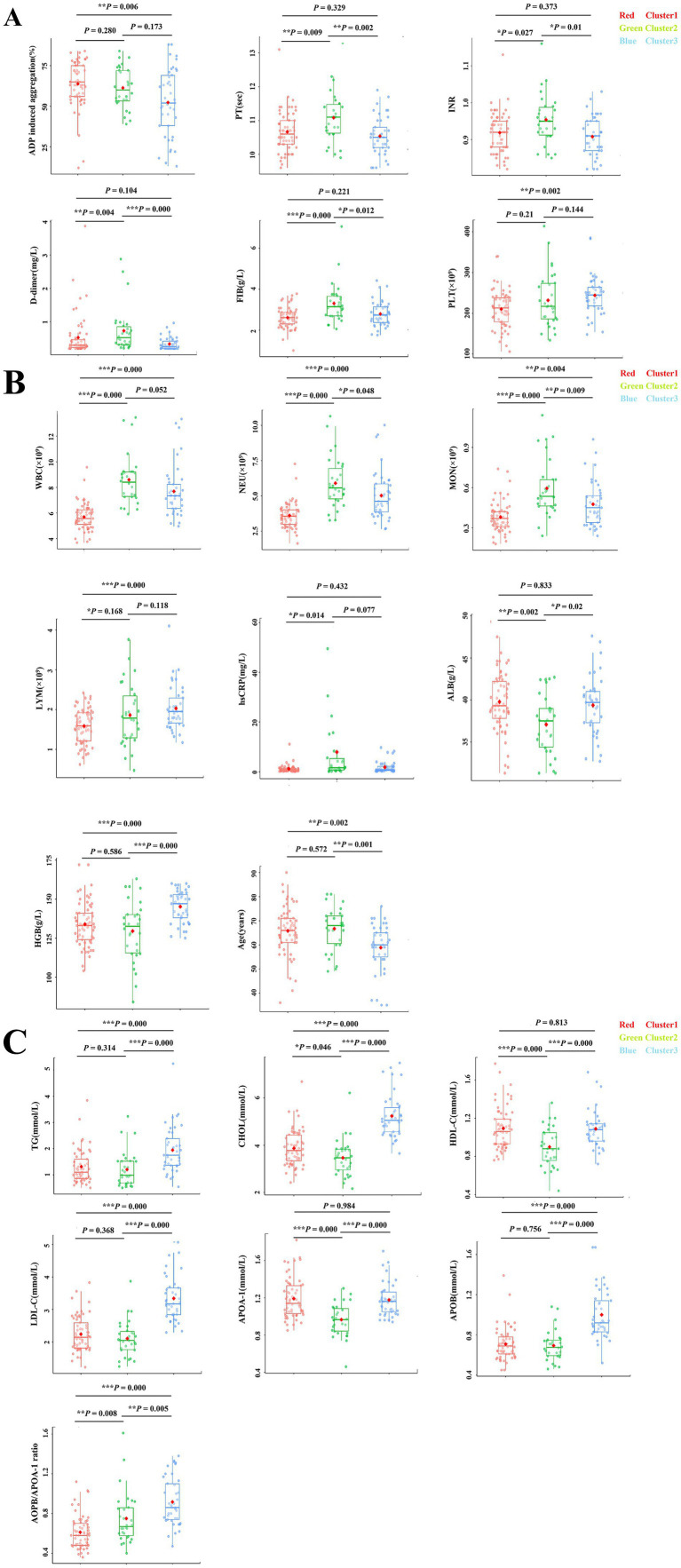
Pairwise comparison of coagulation, metabolic, and inflammatory parameters among ischemic stroke patient clusters. Box plots displayed pairwise comparisons of coagulation, metabolic, and inflammatory parameters among three IS patient subtypes. **(A)** Coagulation parameters including ADP inducing aggregation, PT, INR, D-dimer, FIB, and PLT. **(B)** Systemic inflammatory parameters including WBC, NEU, MON, LYM, hsCRP, ALB HGB, and Age. **(C)** Metabolic parameters including TG, CHOL, HDL-C, LDL-C, APOA-1, APOB, APOB/APOA-1 ratio. Individual data points are superimposed on the box plots to display distribution with each cluster. Significance levels are indicated as *p < 0.05, **p < 0.01, ***p < 0.001.

The heatmap of key variables revealed biochemical and clinical profiles across the three clusters, indicating internal consistency within groups and sharp intergroup differences between clusters (Figure 2), which illustrated overall patterns of laboratory variable distribution across clusters, while detailed comparisons of individual coagulation and metabolic markers were quantified using scatter boxplots (Figure 3) and summarized in Table 1.

**Table 1:** Comparison of hematological and biochemical parameters among the three clusters of ischemic stroke patients.

Variable	Cluster 1 (*n* = 61)	Cluster 2 (*n* = 30)	Cluster 3 (*n* = 41)	*p* value	Cluster 1 vs. 2	Cluster 1 vs. 3	Cluster 2 vs. 3
Age, median (range) (years)	66 (36–90)	68 (49–81)	60 (35–76)	0.001	0.572	0.002	0.001
ADP induced aggregation (%)	65.0 (12.0–84.0)	60.0 (39.0–84.0)	52.0 (13.0–88.0)	0.013	0.280	0.006	0.173
PT, median (range) (s)	10.6 (9.6–13.1)	11.1 (9.9–13.3)	10.5 (9.6–11.9)	0.004	0.009	0.329	0.002
APTT, median (range) (s)	25.5 (19.5–30.5)	25.8 (20.9–29.3)	25.4 (21.5–32.7)	0.824	–	–	–
INR	0.92 (0.82–1.13)	0.95 (0.85–1.16)	0.91 (0.82–1.03)	0.015	0.027	0.373	0.010
FIB, median (range) (g/L)	2.61 (1.01–3.78)	3.145 (2.06–7.06)	2.74 (1.76–4.42)	0.000	0.000	0.221	0.012
TT, median (range) (s)	18.2 (16.5–22.8)	17.6 (14.7–19.0)	18.4 (16.4–49.0)	0.004	0.006	0.418	0.002
D-dimer, median (range) (mg/L)	0.29 (0.19–3.87)	0.52 (0.20–2.88)	0.25 (0.19–0.96)	0.000	0.004	0.104	0.000
GLU, median (range) (mmol/L)	5.57 (2.54–18.28)	5.99 (3.35–19.19)	5.95 (1.59–14.86)	0.151	–	–	–
TG, median (range) (mmol/L)	1.10 (0.50–3.84)	0.98 (0.50–3.23)	1.75 (0.54–5.24)	0.000	0.314	0.000	0.000
CHOL, median (range) (mmol/L)	3.77 (2.42–6.69)	3.465 (2.16–6.21)	5.05 (3.65–7.48)	0.000	0.046	0.000	0.000
HDL-C, median(range)(mmol/L)	1.06 (0.76–1.77)	0.88 (0.44–1.36)	1.08 (0.72–1.68)	0.000	0.000	0.813	0.000
LDL-C, median (range) (mmol/L)	2.14 (1.22–3.83)	2.045 (1.24–3.87)	3.17 (2.29–5.10)	0.000	0.368	0.000	0.000
APOA-1, median (range) (g/L)	1.14 (0.85–1.82)	0.965 (0.46–1.30)	1.16 (0.94–1.70)	0.000	0.000	0.984	0.000
APOB, median (range) (g/L)	0.69 (0.45–1.39)	0.675 (0.46–1.08)	0.92 (0.52–1.67)	0.000	0.756	0.000	0.000
APOB/APOA-1	0.58 (0.36–1.12)	0.67 (0.40–1.61)	0.86 (0.47–1.38)	0.000	0.008	0.000	0.005
ALB, median (range) (g/L)	39.3 (31.3–49.3)	37.5 (31.3–42.7)	39.7 (32.7–47.6)	0.004	0.002	0.833	0.020
HGB, median (range) (g/L)	133.0 (104.0–172.0)	132.5 (84.0–163.0)	147.0 (125.0–160.0)	0.000	0.586	0.000	0.000
WBC, median (range) (×10^9^/L)	5.57 (3.68–9.57)	8.405 (5.92–13.46)	7.34 (4.96–13.34)	0.000	0.000	0.000	0.052
NEU, median (range) (×10^9^/L)	3.56 (1.63–7.27)	5.565 (3.27–10.65)	4.62 (2.65–10.02)	0.000	0.000	0.000	0.048
MON, median (range) (×10^9^/L)	0.37 (0.18–0.74)	0.535 (0.24–1.14)	0.45 (0.24–0.96)	0.000	0.000	0.004	0.009
LYM, median (range) (×10^9^/L)	1.59 (0.62–2.42)	1.785 (0.47–3.76)	1.95 (1.17–4.10)	0.002	0.168	0.000	0.118
PLT, median (range) (×10^9^/L)	213.0 (106.0–340.0)	217.0 (134.0–414.0)	245.0 (148.0–385.0)	0.005	0.210	0.002	0.144
MPV, median (range) (fL)	10.1 (8.2–12.9)	10.2 (8.8–11.7)	9.8 (8.2–11.1)	0.239	–	–	–
PDW, median (range) (fL)	11.4 (7.8–17.4)	11.7 (8.3–14.3)	10.9 (8.0–14.2)	0.239	–	–	–
PCT, median (range) (%)	0.22 (0.11–0.35)	0.215 (0.15–0.39)	0.24 (0.14–0.39)	0.082	–	–	–
hsCRP, median (range) (mg/L)	0.807 (0.124–11.1)	1.615 (0.124–58.90)	0.758 (0.124–9.75)	0.025	0.014	0.432	0.077
HCY median (range) (mmol/L)	12.39 (3.80–49.32)	12.145 (7.39–43.48)	11.33 (5.88–77.13)	0.605	–	–	–

### Demographic and clinical characteristics among the three clusters of ischemic stroke patients

The PCA and clustering analyses confirmed the presence of three biologically distinct subtypes. Comparison of demographic and clinical characteristics further underscored the clinical heterogeneity among the three IS clusters (Table 2). Patients in Cluster 1 and 2 were significantly older (median 66 and 68 years, respectively) than those (median 60 years, *p* = 0.001) in Cluster 3. Females predominated in Cluster 2 (93.3%) and Cluster 3 (75.6%), whereas Cluster 1 included a relatively higher proportion of males (39.3%) (*p* = 0.011). No significant differences were observed in BMI and NIHSS scores at admission.

**Table 2:** Comparison of demographic and clinical characteristics among the three clusters of ischemic stroke patients.

Variable	Cluster 1 (*n* = 61)	Cluster 2 (*n* = 30)	Cluster 3 (*n* = 41)	*p* value
Sex				0.011
Male, *n* (%)	24 (39.3)	2 (6.7)	10 (24.4)	
Female, *n* (%)	37 (60.7)	28 (93.3)	31 (75.6)	
Age, years, median (range)	66 (36–90)	68 (49–81)	60 (35–76)	0.001
BMI, kg/m^2^, median (range)	24.3 (19.6–31.6)	25.3 (21.7–29.8)	24.5 (21.9–28.7)	0.305
NIHSS, median (range)	3 (0–9)	3 (0–10)	2 (0–11)	0.605
Diabetes, *n* (%)	19 (31.1)	17 (56.7)	15 (36.6)	0.066
Hypertension, *n* (%)	39 (63.9)	25 (83.3)	34 (82.9)	0.053
History of stroke, *n* (%)	36 (59.0)	19 (63.3)	13 (31.7)	0.015
Carotid plaque, *n* (%)	41 (67.2)	29 (96.7)	30 (73.2)	0.015
Coronary heart disease, *n* (%)	12 (19.7)	11 (36.7)	4 (9.8)	0.031
Smoking, *n* (%)	7 (11.5)	14 (46.7)	21 (51.2)	0.000
Drinking, *n* (%)	3 (4.9)	7 (23.3)	12 (29.3)	0.011
CYP2C19 phenotype, *n* (%)				0.750
EM	26 (42.6)	9 (30.0)	16 (39.0)	
IM	24 (39.3)	16 (53.3)	19 (46.3)	
PM	11 (18.0)	5 (16.7)	6 (14.6)	

The traditional risk factors for IS were greatest in Cluster 2. Diabetes (56.7%) and hypertension (83.3%) were more frequent in this cluster, although the differences were borderline significant. Cluster 2 also showed the highest prevalence of carotid plaque (96.7%) and coronary artery disease (36.7%), compared with lower rates in Clusters 1 and 3 (*p* < 0.05). A history of prior stroke was more common in Clusters 1 (59.0%) and 2 (63.3%) than in Cluster 3 (31.7%, *p* = 0.016), suggesting a heavier burden of recurrent cerebrovascular events in the older clusters.

Smoking and alcohol consumption were markedly more prevalent in Clusters 2 and 3, with smoking rates of 46.7 and 51.2%, respectively, compared with only 11.5% in Cluster 1 (*p* = 0.000). Similarly, alcohol intake was higher in Clusters 2 and 3 than in Cluster 1 (*p* = 0.011). No significant differences were observed in CYP2C19 metabolizer phenotype among three clusters.

### Hematological and biochemical parameters among the three clusters of ischemic stroke patients

To further interpret the clinical implications of these data driven subtypes, we next compared hematological and biochemical parameters among the three clusters of this cohort IS patients, and significant differences were observed for multiple variables (Table 1), reflecting distinct physiological profiles between the clusters.

Cluster 2 exhibited markedly elevated WBC, NEU and MON compared with Clusters 1 and 3 (*p* = 0.000 for all), accompanied by significantly higher hsCRP levels (*p* = 0.025). In contrast, Cluster 1 showed relatively lower WBC, NEU, MON and hsCRP, suggesting a more favorable inflammatory status. Hemoglobin (HGB) levels were significantly higher in Cluster 3 (*p* = 0.000). These findings indicate a pronounced inflammatory phenotype in Cluster 2.

FIB and D-dimer levels were significantly elevated in Cluster 2 compared with both Cluster 1 and Cluster 3 (*p* = 0.000 for both). Furthermore, PT and INR were prolonged, and TT was shorter in Cluster 2 compared with the other Clusters (*p* = 0.004, *p* = 0.015, and *p* = 0.004, respectively), indicating dysregulated coagulation activity in Cluster 2.

Cluster 3 exhibited higher levels of TG, CHOL, HDL-C, LDL-C, APOB, APOA-1 and APOB/APOA1 ratio compared with Clusters 1 and 2 (*p* = 0.000 for all), consistent with a pro-atherogenic metabolic state in Cluster 3.

Cluster 1 showed higher ADP induced aggregation. And Cluster 1 also exhibited with higher CHOL, HDL-C and APOA-1 but lower APOB/APOA-1ratio and inflammatory markers compared to Cluster 2, indicating more favorable hematological and biochemical profile in Cluster 1.

To summarize the heterogeneity based on hematological, biochemical, and clinical characteristics. Three clusters were proposed nomenclature of low inflammatory ischemic stroke subtype, high inflammatory-hypercoagulable ischemic stroke subtype and metabolic risk ischemic stroke subtype, respectively. Although these statistical differences delineate between three distinct subtypes, their importance lies in the potential clinical implications, as each profile may correspond to specific risk pathways and therapeutic needs.

### Pairwise comparisons of hematological and biochemical parameters among clusters

Pairwise comparisons demonstrated distinct profiles across the three clusters (Table 1, Figure 3). Clusters 1 and 2 included older patients, whereas Cluster 3 was significantly younger. Cluster 2 showed the most adverse phenotype, with elevated WBC, NEU, hsCRP, FIB, and D-dimer, together with prolonged PT/INR and reduced APOA-1(Figures 3A,B), indicating a strong inflammatory-hypercoagulable state. Cluster 1 was a low inflammatory state, characteristic with older age, high ADP induced aggregation and relatively mild inflammation (Figures 3A,B). In contrast, Cluster 3 exhibited a lipid driven dysmetabolic profile, characterized by higher TG, CHOL, HDL-C, LDL-C, APOA-1, APOB and an elevated APOB/APOA1 ratio (Figure 3C), alongside moderate inflammation (Figure 3B). These findings highlight three biologically distinct ischemic stroke subtypes defined by coagulation, inflammatory, and metabolic features.

## Discussion

In this study, we applied an integrative, data driven clustering approach to characterize biological heterogeneity among patients with ischemic stroke using inflammatory, coagulation, and metabolic markers. Three biologically and clinically subtypes were identified, reflecting differential patterns of systemic inflammation, coagulation activity, and metabolic dysregulation. Rather than relying on single biomarkers or predefined clinical categories, this multidimensional strategy highlights the complex interplay among biological pathways that contribute to ischemic stroke heterogeneity.

The first subtype was characterized by relatively low inflammatory activity with lower inflammatory factors. The second subtype displayed a high inflammatory and hypercoagulable phenotype, marked by increased WBC, NEU, hsCRP, FIB, D-dimer, and prolonged PT/INR, alongside a higher prevalence of traditional vascular risk factors such as coronary artery disease and carotid plaque. The third subtype was distinguished primarily by metabolic abnormalities, including elevated lipid profiles, accompanied by intermediate levels of inflammatory.

The identification of a hyperinflammatory-hypercoagulable subtype is consistent with extensive evidence supporting the central role of inflammation and coagulation abnormalities in stroke pathophysiology. Elevated inflammatory markers, such as hsCRP and NEU, have been repeatedly associated with increased stroke risk, infarct severity, and poor prognosis (Elkind, 2010; Kaptoge et al., 2012; Wang et al., 2016). Likewise, coagulation abnormalities, including increased FIB and D-dimer levels, are known contributors to thrombus formation and recurrent ischemic events (Mccabe et al., 2024; Feller et al., 2022). Importantly, our clustering results demonstrate that these inflammatory and coagulation disturbances frequently co-occur within the same individuals, reinforcing the concept that stroke related biological pathways are tightly interconnected rather than operating in isolation (Folsom et al., 2016). The high proportion of elderly woman in Cluster 2 may reflect postmenopausal hormonal changes, which are known to influence inflammatory and coagulation pathways. Estrogen decline is associated with increased cytokine activity, endothelial dysfunction, and hypercoagulability, which may contribute to the observed phenotype.

Our hypercoagulable cluster demonstrated higher FIB and D-dimer levels, consistent with prior evidence (Ageno et al., 2002), but importantly, the clustering approach revealed how these abnormalities co-occur with distinct clinical phenotypes.

The metabolic risk subtype further underscores the importance of dyslipidemia as a key contributor to ischemic stroke. Elevated LDL-C and APOB are strongly linked to atherosclerotic plaque formation and ischemic stroke incidence (Amarenco et al., 2020; Tziomalos et al., 2017). Interestingly, patients in this cluster also exhibited relatively higher HDL-C levels and intermediate inflammatory activation, suggesting that lipid driven vascular risk may manifest through mechanisms partially independent of overt systemic inflammation. These findings highlight the need to consider metabolic, inflammatory, and coagulation processes jointly when characterizing stroke risk profiles.

It is also important to note that several variables contributing to cluster differentiation, including age, sex, smoking status, and lipid levels, are established risk factors for ischemic stroke. Therefore, the identified clusters should not be interpreted as purely novel molecular endotypes. Rather, they represent composite biological–clinical phenotypes that capture the co-occurrence and interaction of traditional risk factors with inflammatory, coagulation, and metabolic dysregulation. This integrative pattern-based stratification may better reflect real-world patient heterogeneity than analyses focusing on isolated variables.

The novelty of our study lies in the simultaneous incorporation of inflammatory, coagulation, and metabolic markers into an unsupervised clustering framework to capture complex interactions. This integrative approach provides a more refined understanding of ischemic stroke heterogeneity than single marker analyses. By contrast, our approach identified composite subtypes that reflect the interplay among multiple biological processes, a strategy increasingly used in cardiovascular and neurological research (Shah et al., 2015).

From a methodological perspective, the use of unsupervised clustering allowed for the identification of patient subgroups without imposing predefined assumptions. Cluster validity and stability analyses supported the three-cluster solution, while PCA was used primarily for visualization rather than as a determinant of cluster structure. Nevertheless, the modest proportion of variance explained by the first two principal components underscores that ischemic stroke heterogeneity cannot be fully captured in low-dimensional space and should be interpreted within the context of the full multivariate dataset.

Although these findings provide biological insight, several limitations of this study must be acknowledged. First, this was a retrospective, single-center analysis with a relatively modest sample size, which may limit generalizability. Future studies with larger cohorts are needed for validation. Second, and more importantly, longitudinal clinical outcomes such as functional status, stroke recurrence, and mortality were not available. As a result, the identified ischemic stroke subtypes should be interpreted as biologically distinct phenotypes rather than prognostically validated categories. The potential clinical and therapeutic implications discussed herein remain hypothesis generating and require confirmation in prospective studies with long-term follow-up. Third, potential confounders such as genetic predisposition or imaging biomarkers were not included. Finally, while clustering revealed distinct subgroups, external validation in independent cohorts is necessary to confirm reproducibility.

Our findings indicate that a specific patient subtype may harbor biological features relevant to differential treatment responses, warranting further investigation. However, this observational analysis is preliminary and does not support specific treatment recommendations. Future research should aim to validate these biomarker-defined phenotypes in larger, multicenter cohorts and to integrate additional data modalities, such as imaging markers, genetic information, and longitudinal outcomes.

## Conclusion

Data-driven clustering revealed three biologically distinct ischemic stroke phenotypes defined by inflammation, coagulation, and metabolic profiles. This stratification highlights the multidimensional heterogeneity of ischemic stroke and provides a framework for future studies aimed at validating prognostic significance and treatment responsiveness in prospective cohorts.

## Data Availability

The original contributions presented in the study are included in the article/Supplementary material, further inquiries can be directed to the corresponding author.

## Glossary

ADP induced aggregation: adenosine diphosphates induce aggregation
PT: prothrombin time
APTT: activated partial thromboplastin time
INR: international normalized ratio
FIB: fibrinogen
TT: thrombin time
GLU: glucose
TG: triglyceride
CHOL: cholesterol
HDL-C: high density lipoprotein cholesterol
LDL-C: low density lipoprotein cholesterol
APOA-1: apolipoprotein A-1
APOB: apolipoprotein B
ALB: albumin
HGB: hemoglobin
WBC: white blood cell
NEU: neutrophil
MON: monocyte
LYM: lymphocyte
PLT: platelet
MPV: mean platelet volume
PDW: platelet distribution width
PCT: plateletcrit
hsCRP: high sensitivity C reactive protein
HCY: homocysteine
PCA: principal component analysis
BMI: body mass index
NIHSS: National Institutes of Health Stroke Scale
