# Supplementary figures and images for: Identification of ischemic stroke subtypes defined by inflammation, coagulation, and metabolic profiles
Source: Front Artif Intell. 2026 Mar 10;9:1776891. doi: 10.3389/frai.2026.1776891 (PMC13008919; doi:10.3389/frai.2026.1776891)

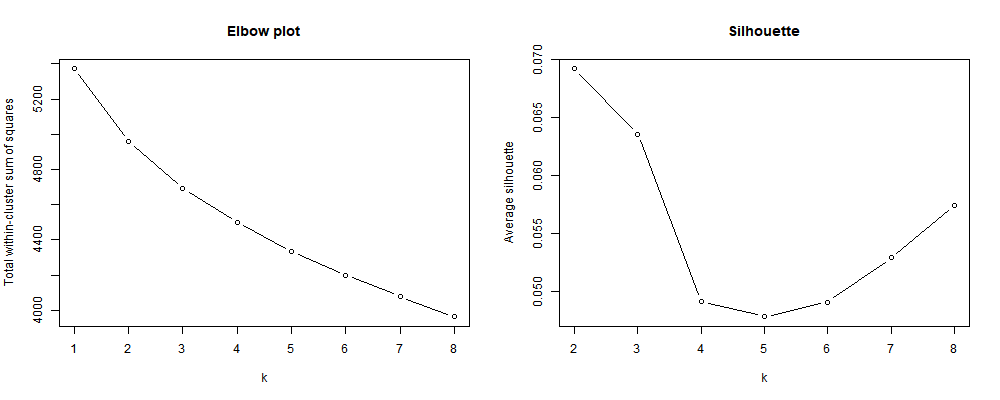

Supplement: Supplementary file 1 [file Image_1.PNG]
